# Estimation of Effective Day Length at Any Light Intensity Using Solar Radiation Data

**DOI:** 10.3390/ijerph8114272

**Published:** 2011-11-10

**Authors:** Masana Yokoya, Hideyasu Shimizu

**Affiliations:** 1 Shimonoseki Junior College, 1-1 Sakurayama-cho, Shimonoseki City, 750-8508, Japan; 2 Toshiwa-kai Hospital, 5-8-1 Kanayama, Nakaku, Nagoya City, 460-0022, Japan; E-Mail: info@toshiwa-kai.or.jp

**Keywords:** solar radiation, effective day length, luminous efficiency, light intensity, circadian rhythm

## Abstract

The influence of day length on living creatures differs with the photosensitivity of the creature; however, the possible sunshine duration (*N_0_*) might be an inadequate index of the photoperiod for creatures with low light sensitivity. To address this issue, the authors tried to estimate the effective day length, *i.e.*, the duration of the photoperiod that exceeds a certain threshold of light intensity. Continual global solar radiation observation data were gathered from the baseline surface radiation network (BSRN) of 18 sites from 2004 to 2007 and were converted to illuminance data using a luminous efficiency model. The monthly average of daily photoperiods exceeding each defined intensity (1 lx, 300 lx, … 20,000 lx) were calculated [defined as *Ne*_(_*_lux_*_)_]. The relationships between the monthly average of global solar radiation (*Rs*), *N_0_*, and *Ne*_(_*_lux_*_)_ were investigated. At low light intensity (<500 lx), *Ne*_(_*_lux_*_)_ were almost the same as *N_0_*. At high light intensity (>10,000 lx), *Ne*_(_*_lux_*_)_ and *Rs* showed a logarithmic relationship. Using these relationships, empirical models were derived to estimate the effective day length at different light intensities. According to the validation of the model, the effective day length for any light intensity could be estimated with an accuracy of less than 11% of the mean absolute percentage error (MAPE) in the estimation of the monthly base photoperiod. Recently, a number of studies have provided support for a link between day length and some diseases. Our results will be useful in further assessing the relationships between day length and these diseases.

## 1. Introduction

Day length is an important environmental factor that affects all living things. However, these influences depend on the species, namely, the critical day length or the light sensitivity is different for each creature. For example, the sensitivity of the human circadian melatonin rhythm is not the same as the photosensitivity of flower bud formation in plants. Compared to plants, relatively high light intensities (50–600 lx) can induce considerable phase shifts in the human circadian melatonin rhythm [1]. In humans, modulation of circadian rhythms by light is thought to be mediated primarily by melanopsin containing retinal gangrion cells. Melanopsin cells are intrinsically blue light sensitive, and one cannot make melatonin when blue light levels are too high [2–4].

The photoperiodic response of plants is very sensitive to light conditions, so possible sunshine duration (*N_0_*) can be employed as an index of the day length [5]; however this index is ineffective for many other creatures with low light sensitivity. For such creatures, the duration of photoperiod that exceeds the threshold of sensitivity is different from the possible sunshine duration, and this duration varies depending on the weather conditions. However, there is no available climatic element that can serve as an index of these durations. *N_0_* or sunshine duration time (*n*) is ineffective as indexes of these durations.

To address this issue, it is necessary to estimate the effective day length, *i.e.*, the duration of photoperiod exceeding the threshold of light intensity. In this study, the authors have attempted to derive a formula for estimating the effective day length at any light intensity using the existing weather data obtained from meteorological stations.

The ultimate aim of this paper is biological or medical application. The seasonal change in circadian rhythm phase is generally due to seasonal variations of photoperiod. Recently, a number of studies have provided support for a link between the altered circadian phase shifts associated with 24-h lifestyle and obesity [6], and there are documented effects of day length, light at night, and melatonin production on cancer rates [7–13]. In order to make clear the relationship between day length and these diseases, it is important to assess the seasonal variations of the effective day length. So here, we present the estimation results of the monthly average of effective day length at light intensities of 1–20,000 lx.

## 2. Materials and Methods

### 2.1. BSRN Data

For the purpose of the study, it is desirable to use daylight illuminance data measured in various fields around the World. However, the field measurement of daylight illuminance on a global scale has not yet been conducted. Therefore, we used solar radiation data as input data. Continual global solar radiation (*Rs*) observation data were gathered from the baseline surface radiation network (BSRN) of 18 stations located from 50°N to 50°S over a period of 4 years between 2004 and 2007 [14]. The site locations are listed in Table 1. In this study, only data from low- to mid-latitude sites were gathered, because data from high-latitude sites can interfere with the analysis because of the weak and long day light in summer. Solar radiation measurements were obtained from the BSRN system, which provides 1- or 3-min averages of the global solar radiation. In this study, only days for which 24-h observations were performed without missing any data were considered.

### 2.2. Definition of Effective Day Length

The solar radiation data were converted to light intensity data using a Igawa’s luminous efficiency model [15,16] that calculates the daylight intensity using solar radiation and solar altitude data. This model is well known for its validity. The luminous efficiency model is:

(1) $$\begin{array}{l} Evg=(8.86\times h+210.12)\times {Rs}^{0.9}\\ +(-10.98\times h^{4}+54.16\times h^{3}-102.31\times h^{2}+90.21\times h-29.24)\times {Rs}^{1.1} \end{array}$$

where *Evg* is light intensity (global horizontal illuminance; lx), *Rs* is global horizontal solar radiation (W m^−2^), *h* is solar zenith angle (rad).

The photoperiods exceeding each defined light intensity (1 lx, 300 lx, 500 lx, 1,000 lx, …, 20,000 lx) were integrated at daily intervals. Next monthly averages of these photoperiods were calculated. In this study, this photoperiod is defined as the effective day length [*Ne_(lux)_*], where *lux* is the threshold of each light intensity indicated by the global horizontal illuminance.

### 2.3. Data Analysis

The monthly average of daily extraterrestrial radiation *Ra* (MJ m^−2^ day^−1^) and possible sunshine duration *N_0_* (h day^−1^) were calculated following the guidelines of Darula *et al*. [17], and the monthly average of daily amount of global solar radiation *Rs* (MJ m^−2^day^−1^) was calculated using the BSRN data. In order to derive the model that estimates the effective day length at any light intensity, the relationships between the monthly averages of *Ra*, *N*, *Rs*, and *Ne_(lux)_* (h day^−1^) were investigated. In this case, only months when more than 20 days were observed without missing any data were considered. The numbers of analyzed months are shown in Table 1.

To determine the coefficients of the general regression equation, the following steps were taken. First, the regression coefficients between the monthly averages of *Ra*, *N*, *Rs*, and *Ne_(lux)_* were calculated at the defined light intensity. Then, the trends of the coefficients for all the light intensities were investigated, and a generalized model that could be applied to any light intensity was derived.

### 2.4. Model Validation

Global solar radiation measurement data from Tateno (Japan), Chesapeake Light (USA), and Payerne (Switzerland) for 2008 were gathered from the baseline surface radiation network (BSRN), and were converted to light intensity data using an Igawa’s luminous efficiency model. We calculated monthly averages of effective day length from these data and used for a consistency check. Additionally, in order to verify the model, direct measurements of illuminance data from Vaulx-en-Velin (France) for 2008–2010 were obtained [18]. Monthly averages of the effective day length were calculated, and the estimated effective day length and the observed one were compared. Then, the root mean square error (RMSE) and mean absolute percentage error (MAPE) were calculated. The MAPE was calculated as follows:

(2) $$\frac{1}{\text{n}}\sum_{1}^{\text{n}}|\frac{\text{Observed}-\text{Estimated}}{\text{Estimated}}|\times 100$$

The site locations used for the validation are listed in Table 2.

Statistical analyses were carried out using R version 2.7.1 [19].

## 3. Results

### 3.1. Relationships between Monthly Average of Effective Day Length and Other Climatic Elements

**Figure 1:**
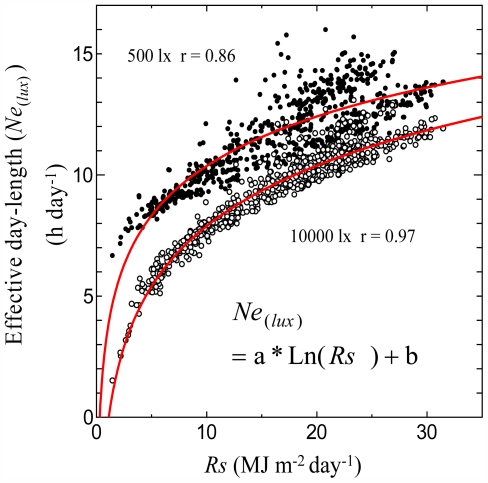
Relationship between monthly average of effective day length (*Ne_(lux)_*) and monthly average of global solar radiation (*Rs*).

Figure 1 shows the relationships between the monthly average of *Ne_(500)_* (h day^−1^) and the monthly average of *Rs* (MJ m^−2^ day^−1^) and between the monthly average of *Ne_(10000)_* and monthly average of *Rs*. At low light intensity (500 lx), the relationships were not clear. At low light intensity, the *Ne_(lux)_* values were almost the same as the monthly averages of *N_0_* (h day^−1^). This means effective day length at low light intensities will be free of the influence of any weather conditions. At high light intensity (10,000 lx), the relationships between *Ne_(lux)_* and *Rs* were clear and a logarithmic curve was well fitted.

**Figure 2:**
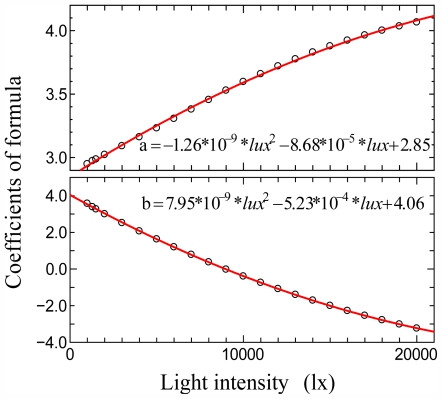
Trend of coefficients of Equation (3) for all light intensities.

The trend of the coefficients was investigated for all light intensities (Figure 2), and a generalized formula that could be applied to any light intensity was derived:

(3) $${Ne}_{\left(lux\right)}=\text{a}\times \text{Ln}(Rs)+\text{b}$$

(4) $$\text{a}=-1.07\times 10^{-9}\times {\mathrm{lux}}^{2}+8.35\times 10^{-5}\times lux+2.91$$

(5) $$\text{b}=9.21\times 10^{-9}\times {\mathrm{lux}}^{2}-5.61\times 10^{-4}\times lux+4.19$$

**Figure 3:**
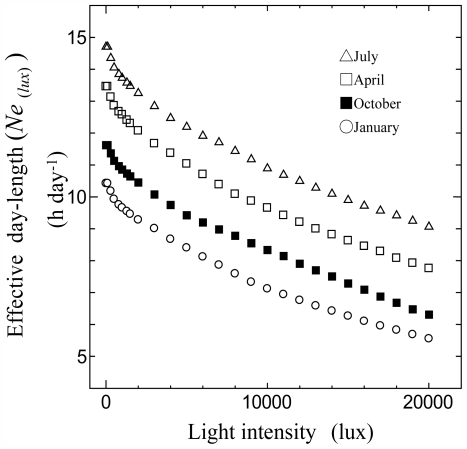
Relationships between monthly average of effective day length (*Ne_(lux)_*) and light intensity at Tateno station in 2008.

Figure 3 shows the relationships between the monthly average of the effective day length (*Ne_(lux)_*) and the light intensity at Tateno station in 2008. Because *Ne_(lux)_* varies inversely with light intensity in relation to the monthly average of *N_0_*, at low light intensity of less than 10,000 lx, the following formula can be applied:

(6) $${Ne}_{\left(lux\right)}=(N_{0}-{Ne}_{\left(10000\right)})\times ((10000-lux)/10000)+{Ne}_{\left(10000\right)}$$

where *N_0_* is the monthly average of possible sunshine duration (h day^−1^). Equation (6) shows the linear interpolation between the possible sunshine duration and the monthly average of effective day length at light intensity of 10,000 lx. As we did not observe a trend for the change in the coefficients in Figure 3, we considered a simple linear relationship; however, the error caused by this is probably small. There was no significant relation between *Ra*, *n*, and *Ne_(lux)_*.

### 3.2. Consistency Check

**Figure 4:**
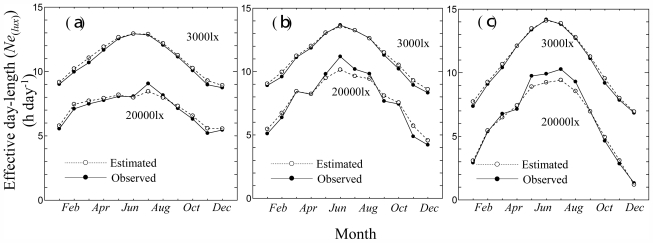
Seasonal transition of observed and estimated monthly mean effective day length (*Ne_(lux)_*) at 3,000 lux (upper) and 20,000 lux (lower) for Tateno (**a**), Chesapeake Light (**b**) and Payerne (**c**) in 2008.

In order to check the model, global solar radiation measurements data from Tateno (Japan), Chesapeake Light (USA), and Payerne (Switzerland) for 2008 were converted to light intensity data using a luminous efficiency model and monthly average of effective day length were derived and used for a consistency check. Figure 4 shows the seasonal transition of observed and estimated monthly mean effective day length [*Ne_(lux)_* h day^−1^] at 3,000 lx (upper) and 20,000 lx (lower) at Tateno (a), Chesapeake Light (b) and Payerne (c). At 3000 lx, the estimation was carried using Equations (3)–(6); 20,000 lx, Equations (3)–(5). The RMSE calculated through the year was 0.44 h day^−1^ (MAPE: 3.9%) at 3,000 lx and 0.29 h day^−1^ (MAPE: 3.7%) at 20,000 lx in Tateno. Likewise, the RMSE was 0.36 h day^−1^ (MAPE: 3.3%) at 3,000 lx and 0.47 h day^−1^ (MAPE: 5.7%) at 20,000 lx in Chesapeake Light, and the RMSE was 0.51 h day^−1^ (MAPE: 5.0%) at 3,000 lx and 0.44 h day^−1^ (MAPE: 5.9%) at 20,000 lx in Payerne.

**Figure 5:**
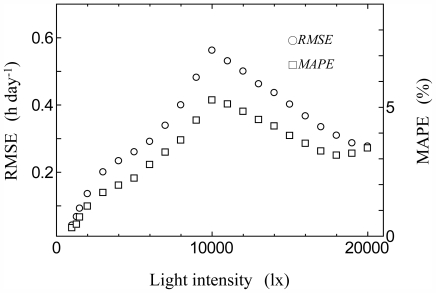
Transition of RMSE and MAPE at all light intensities in estimation of monthly base photoperiod at Tateno station in 2008.

Figure 5 shows the transition of RMSE and MAPE at all the light intensities for the monthly base estimation at Tateno station in 2008. The RMSE and MAPE increased at around 10,000 lx, with a similar trend in Chesapeake Light and Payerne. This is because, at low light intensities, the effective day length is almost the same as the possible sunshine duration. Hence, at high light intensities, the logarithmic curve fitted well. The maximum value of RMSE at all the light intensities was 0.56 h day^−1^, and the maximum value of MAPE was 5.2% at Tateno.

In Figure 5, the transitions of RMSE and MAPE are discontinuous around 10,000 lx. This is because a different equation was used to estimate the effective day length. We decided to set a limit on the choice of equations from the fitting conditions of the equation or the results of validation, but this sometimes causes a discontinuity in the estimation. The maximum value of RMSE at all the light intensities was 0.53 h day^−1^ and 0.82 h day^−1^ in Chesapeake Light and Payerne stations, respectively. The maximum value of MAPE was 5.7% and 6.8% for Chesapeake Light and Payerne stations, respectively.

**Figure 6:**
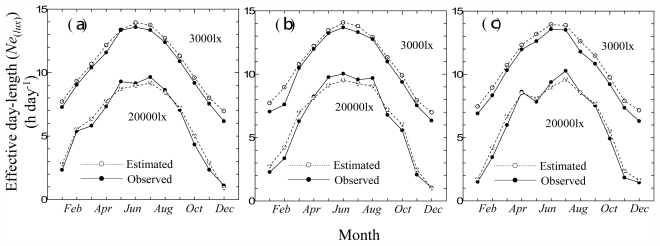
Seasonal transition of observed and estimated monthly mean effective day length (*Ne_(lux)_*) at 3,000 lux (upper) and 20,000 lux (lower) for Vaulx-en-Velin in 2008 (**a**), 2009 (**b**) and 2010 (**c**).

Figure 6 shows the seasonal transition of observed and estimated monthly mean effective day length [*Ne_(lux)_* h day^−1^] at 3,000 lx (upper) and 20,000 lx (lower) at Vaulx-en-Velin (France) station in 2008 (a), 2009 (b) and 2010 (c).

At 3000 lx, the estimation was carried using Equations (3)–(6); 20,000 lx, Equations (3)–(5). At 3,000 lx, the estimated values slightly exceeded the observed values throughout the year in all years. This was probably due to the linear interpolation of Equation (6). The RMSE calculated through the year was 0.43 h day^−1^ (MAPE: 4.4%) at 3,000 lx and 0.42 h day^−1^ (MAPE: 9.3%) at 20,000 lx in 2008. Likewise, the RMSE was 0.57 h day^−1^ (MAPE: 5.5%) at 3,000 lx and 0.52 h day^−1^ (MAPE: 10.1%) at 20,000 lx in 2009, and the RMSE was 0.57 h day^−1^ (MAPE: 6.0%) at 3,000 lx and 0.45 h day^−1^ (MAPE: 9.9%) at 20,000 lx in 2010.

**Figure 7:**
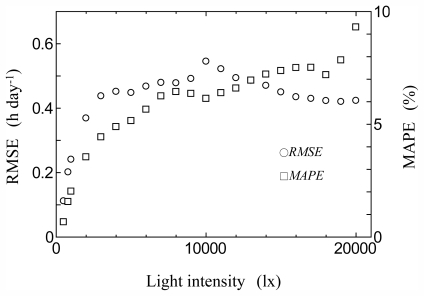
Transition of RMSE and MAPE at all light intensities in estimation of monthly base photoperiod at Vaulx-en-Velin station in 2008.

Figure 7 shows the transition of RMSE and MAPE calculated through the year at all the light intensities for the monthly base estimation at Vaulx-en-Velin station in 2008. The RMSE increased at around 10,000 lx, with a similar trend in the consistency check. The maximum value of RMSE at all the light intensities was 0.54 h day^−1^ (at 10,000 lx), and the maximum value of MAPE was 9.3% (at 20,000 lx) in 2008. The MAPE increased at high light intensities, as the actual effective day length was so short. The maximum value of RMSE at all the light intensities was 0.65 h day^−1^ and 0.52 h day^−1^ in 2009 and 2010, respectively. The maximum value of MAPE was 10.1% and 9.9% in 2009 and 2010, respectively.

As a result, the effective day length at any light intensity could be estimated with an accuracy of less than 0.9 h day^−1^ of RMSE (11% of MAPE) in the estimation of the monthly base photoperiod.

## 4. Discussion

We have developed a simple method for estimating the effective day length at any light intensity using solar radiation data. From the relationships between the effective day length and possible sunshine duration at low light intensity (<500 lx), the effective day length was found to be almost equal to the possible sunshine duration. Considering the minimum light intensities that are photoperiodically effective, at low light intensities the accuracy of our estimation was not always superior to the minimum detectable sensitivity of living creatures.

However, in humans, 50–600 lx of light intensities can induce considerable phase shifts in the circadian melatonin rhythm [1], and in general, more than 1,000 lx of light intensities are needed to treat sleep-wake rhythm disorders [20]. In addition, people who spend long hours indoors will be affected by more stronger light [21,22]. Actually, more than 1,000 lx of outdoor illumination is important to evaluate indoor illumination that induces phase shifts in the human circadian melatonin rhythm. According to the validation of the model, the effective day length at relatively high light intensity (>1,000 lx) could be estimated with an accuracy of less than 11% of the mean absolute percentage error (MAPE) in the estimation of the monthly base photoperiod. Because our model was derived from the luminance data converted from the solar radiation data, potential biases and errors specific to the luminous efficiency model might be built in the model; however, the error caused by this is probably small. It is considered that our model is broadly applicable to the analysis of the human light environment.

Additionally, considering that the error in estimating the daily solar radiation from the sunshine duration is about 10% [23–25], the accuracy of our model appears to be at a reasonable level. However, the results were obtained empirically and they have not yet been confirmed theoretically. To enhance the accuracy of this model, it is necessary to investigate the relationships between latitude, sun elevation, and cloud cover.

As a result, the effective day length at high light intensity was found to be described by the simple increasing function of the solar radiation. This means the distribution of the effective day length could be similar to the distribution of the solar radiation.

Recently, a number of studies have provided support for a link between the altered circadian phase shifts associated with 24-h lifestyle and obesity [6]. Additionally, there are documented effects of day length, light at night, and melatonin production on cancer rates [7–13]. Our results will be useful in further assessing the relationships between day length and these diseases. Further research is required to identify the association between these climatic factors and diseases.

## Figures and Tables

**Table 1:** Location of BSRN stations and analyzed months without any missing data between 2004 and 2007.

	Observation Points	Position		Number of Observed Months			

		Lat.	Lon.	2004	2005	2006	2007
1	Alice Springs	−23.8	133.9	12	12	12	12
2	Bondville	40.1	−88.4	12	12	12	12
3	Boulder Surfrad	40.1	−105.2	12	12	11	12
4	Carpentras	44.1	5.1	12	12	11	12
5	Cocos Island	− 12.2	96.8	-	-	-	11
6	Darwin	−12.4	130.9	6	9	7	7
7	Desert Rock	36.6	−116.0	12	12	12	12
8	Fort Peck	48.3	−105.1	12	12	12	12
9	Goodwin Creek	34.3	−89.9	10	12	12	12
10	Lauder	−45.1	169.7	12	12	12	12
11	Momote	−2.1	147.4	-	1	2	1
12	Nauru Island	−0.5	166.9	-	-	3	3
13	Rock Springs	40.7	−77.9	11	12	12	12
14	Sede Boqer	30.0	34.0	2	3	10	11
15	Sioux Falls	43.7	−96.6	12	12	12	11
16	São Martinho da Serra	− 29.4	− 53.8	-	-	-	12
17	Tamanrasset	22.8	5.5	12	12	12	12
18	Xianghe	39.8	116.7	-	2	10	8

**Table 2:** Location of stations used for validation.

	Observation Points	Position		Observed Year

		Lat.	Lon.	
1	Tateno (Japan)	36.1	14.3	2008
2	Chesapeake Light (USA)	36.9	−75.7	2008
3	Payerne (Switzerland)	46.8	6.9	2008
4	Vaulx-en-Velin (France)	45.8	4.9	2008–2010

## References

[1] Boivin D.B., Duffy J.F., Kronauer R.E., Czeisler C.A. (1996). Dose-response relationships for resetting of human circadian clock by light. Nature.

[2] Brainard G.C., Hanifin J.P., Greeson J.M., Byrne B., Glickman G., Gerner E., Rollag M.D. (2001). Action spectrum for melatonin regulation in humans: Evidence for a novel circadian photoreceptor. J. Neurosci.

[3] Gooley J.J., Rajaratnam S.M., Brainard G.C., Kronauer R.E., Czeisler C.A., Lockley S.W. (2010). Spectral responses of the human circadian system depend on the irradiance and duration of exposure to light. Sci. Transl. Med.

[4] Thapan K., Arendt J., Skene D.J. (2001). An action spectrum for melatonin suppression: Evidence for a novel non-rod, non-cone photoreceptor system in humans. J. Physiol.

[5] McClung C.R. (2006). Plant circadian rhythms. Plant Cell.

[6] Bray M.S., Young M.E. (2007). Circadian rhythms in the development of obesity: Potential role for the circadian clock within the adipocyte. Obes. Rev.

[7] Borisenkov M.F., Bazhenov S.M. (2005). Seasonal patterns of breast tumor growth in Far North residents. Vopr. Onkol.

[8] Oh E.Y., Ansell C., Nawaz H., Yang C.H., Wood P.A., Hrushesky W.J. (2010). Global breast cancer seasonality. Breast Cancer Res. Treat.

[9] Reiter R.J., Tan D.X., Erren T.C., Fuentes-Broto L., Paredes S.D. (2009). Light-mediated perturbations of circadian timing and cancer risk: A mechanistic analysis. Integr. Cancer Ther.

[10] Yang X., Wood P.A., Ansell C.M., Quiton D.F., Oh E.Y., Du-Quiton J., Hrushesky W.J. (2009). The circadian clock gene Per1 suppresses cancer cell proliferation and tumor growth at specific times of day. Chronobiol. Int.

[11] Kegel M., Dam H., Ali F., Bjerregaard P. (2009). The prevalence of seasonal affective disorder (SAD) in Greenland is related to latitude. Nord. J. Psychiatry.

[12] Monteleone P., Martiadis V., Maj M. (2011). Circadian rhythms and treatment implications in depression. Prog. Neuropsychopharmacol. Biol. Psychiatry.

[13] Magnusson A., Partonen T. (2005). The diagnosis, symptomatology, and epidemiology of seasonal affective disorder. CNS Spectr.

[14] World Radiation Monitoring Center, Baseline Surface Radiation Network (BSRN) Database Website http://www.bsrn.awi.de/en/home/.

[15] Igawa N., Nakamura H., Goto K., Shimosaki S. (1999). A method for the estimation of the solar illuminance based upon the solar irradiance. J. Arch. Environ. Eng.

[16] Igawa N. (2009). An examination of the luminous efficacy of daylight. 6th Lux Pacifica.

[17] Darula S., Kittler R., Kambezidis H., Bartzokas A. (2000). Guidelines for More Realistic Daylight Exterior Conditions in Energy Conscious Design. Computer Adaptation and Examples, SK-GR 013/1998.

[18] Vaulx-en-Velin Station Database Website http://idmp.entpe.fr/vaulx/mesfr.htm.

[19] R Foundation for Statistical Computing Website http://www.r-project.org.

[20] Sack R.L., Auckley D., Auger R.R., Carskadon M.A., Wright K.P., Vitiello M.V., Zhdanova I.V. (2007). Circadian rhythm sleep disorders: Part I, basic principles, shift work and jet lag disorders. Sleep.

[21] Cole R.J., Kripke D.F., Wisbey J., Mason W.J., Gruen W., Hauri P.J., Juarez S. (1995). Seasonal variation in human illumination exposure at two different latitudes. J. Biol. Rhythms.

[22] Hebert M., Dumont M., Paquet J. (1998). Seasonal and diurnal patterns of human illumination under natural conditions. Chronobiol. Int.

[23] Chegaar M., Lamri A., Chibani A. (1998). Estimating global solar radiation using sunshine hours. Rev. Energ. Ren.

[24] Paltineanu Cr., Mihailescu I.F., Torica V., Albu A.N. (2002). Correlation between sunshine duration and global solar radiation in south-eastern Romania. Int. Agrophysics.

[25] Yang Q., Tsukamoto O. (2009). Estimation of daily solar radiation from sunshine duration in Ningxia region, China. Okayama Univ. Earth Sci. Rep.

